# Age at cardiovascular disease onset, dementia risk, and the role of lifestyle factors

**DOI:** 10.1002/alz.13562

**Published:** 2023-12-12

**Authors:** April C. E. van Gennip, Thomas T. van Sloten, Aurore Fayosse, Séverine Sabia, Archana Singh‐Manoux

**Affiliations:** ^1^ Epidemiology of Ageing and Neurodegenerative Diseases, Inserm U1153 Université Paris Cité Paris France; ^2^ Department of Internal Medicine Maastricht University Medical Centre Maastricht The Netherlands; ^3^ School for Cardiovascular Diseases CARIM Maastricht University Maastricht The Netherlands; ^4^ Department of Vascular Medicine University Medical Center Utrecht Utrecht The Netherlands; ^5^ Faculty of Brain Sciences University College London London UK

**Keywords:** age at onset, cardiovascular disease, dementia, epidemiology, lifestyle factors

## Abstract

**INTRODUCTION:**

We first examined the role of age at cardiovascular disease (CVD) onset for incident dementia, and then examined whether lifestyle factors at guideline‐recommended levels in individuals with CVD mitigates dementia risk.

**METHODS:**

We used population‐based data (Whitehall II: n = 10,308/baseline 1985–1988/examinations every 4–5 years). Lifestyle factors (non‐smoking, body mass index [BMI], physical activity, diet) were extracted post‐CVD.

**RESULTS:**

Over a median of 31.6 years, 3275 (32.1%) developed CVD. At age 70, risk of dementia was higher in individuals with CVD onset before (hazard ratio [HR] of incident dementia for participants with CVD before age 60, using participants without CVD at age 70 as the reference: 1.56, 95% confidence interal [CI] 1.18–2.08) but not after 60 years. In participants with CVD, a greater number of lifestyle factors at recommended levels post‐CVD was associated with a lower dementia risk (per lifestyle factor at recommended level HR: 0.73, 95% CI 0.59–0.92).

**DISCUSSION:**

Our results suggest that early onset CVD is associated with a higher dementia risk at older ages. In those with CVD, the dementia risk was lower if lifestyle factors are at recommended levels following CVD diagnosis.

**Highlights:**

- CVD in midlife but not in late life is associated with a higher risk of dementia.
- Dementia risk in CVD patients is lower if their lifestyle factors are at recommended levels.
- These findings provide evidence to promote CVD prevention in midlife or earlier.
- Study findings also show the importance of a healthy lifestyle in those with CVD.

## 1 BACKGROUND

Dementia currently affects more than 55 million people worldwide, and this number is expected to reach 139 million in the year 2050.^1^ After coronary heart disease, dementia is the second leading cause of death in high‐income countries.^2^ Curative solutions for dementia remain elusive, making it urgent to identify potential modifiable targets for prevention of dementia.

Dementia is a progressive disorder known to have a long preclinical period,^3^ making it important for studies on risk factors for dementia to investigate how exposures over the life course shape risk of late‐life dementia.^4^ Recent observational studies have found obesity,^5^ hypertension,^6^ and type 2 diabetes^7^ in midlife rather than late‐life to be associated with an increased risk of dementia. There is also evidence of an association between cardiovascular disease (CVD) and dementia in population‐based studies; these include studies showing higher dementia risk in individuals with coronary heart disease,^8^ stroke,^9^ atrial fibrillation,^10^ and heart failure.^8^ Age is a strong risk factor for both CVD and dementia.^11^ However, most studies included in these meta‐analyses were not able to examine the importance of age at CVD onset for dementia,^8^, ^9^, ^10^ because much of the evidence is based on studies in older adults (≥60 years at study recruitment) with a relatively short follow‐up time (typically <15 years). Therefore, the extent to which early rather than late‐onset CVD is associated with risk of dementia remains unclear.

Care of patients with CVD involves preventive drug therapy and promotion of a healthy lifestyle.^12^, ^13^, ^14^ Secondary prevention in CVD patients is important because it has been proven to be effective in reducing the risk of mortality and recurrence of CVD events.^12^, ^13^, ^14^ In addition, secondary prevention may also affect the risk of old age outcomes such as dementia, given the increase in life expectancy in participants with CVD.^11^ There is also evidence to suggest that the modification of multiple lifestyle factors (e.g., smoking, high body mass index, physical inactivity, and unhealthy diet) might reduce the risk of dementia in the general population.^15^, ^16^ The extent to which the risk of dementia associated with CVD may be mitigated by multifactorial lifestyle modification remains largely unknown. Only one previous study examined that issue, but the focus of that study was atrial fibrillation alone, and the follow‐up was only 3 years.^17^

**RESEARCH IN CONTEXT**

1. **Systematic review**: We reviewed the literature using traditional sources (e.g., PubMed), abstracts, and presentations. Although there is evidence for an association between cardiovascular disease (CVD) and dementia, the importance of age at CVD onset for dementia and the extent to which CVD‐associated dementia risk may be mitigated by multifactorial lifestyle modification after CVD diagnosis remains largely unknown.
2. **Interpretation**: Our findings indicate that midlife (before age 60) but not late‐life CVD is associated with a higher dementia risk. In CVD, dementia risk is lower when lifestyle factors post‐CVD are at guideline‐recommended levels. These findings provide evidence to promote CVD prevention early in life and to encourage healthy lifestyle in CVD.
3. **Future directions**: Further research is needed to replicate our findings and to develop policies for primary CVD prevention in midlife or earlier to mitigate dementia risk. In addition, trials are needed to determine whether lifestyle interventions in people with CVD reduces their dementia risk at older ages.

Using data from the Whitehall II study with a follow‐up spanning from midlife to old age, the aims of the present study were: (1) to examine the association between age at CVD onset and incident dementia and (2) to examine whether lifestyle factors meeting guideline‐recommended levels in individuals with CVD affects the risk of dementia.

## 2 METHODS

### 2.1 Study design

The Whitehall II study is an ongoing prospective cohort study established in 1985–1988 among 10,308 participants employed in London‐based government departments, aged 35–55 years.^18^ The baseline examination consisted of a self‐administrated questionnaire and a structured clinical evaluation composed of measures of anthropometric, cardiovascular, and metabolic risk factors and diseases. Since baseline, follow‐up clinical examinations were completed approximately every 4–5 years (1991–1993, 1997–1999, 2002–2004, 2007–2009, 2012–2013, 2015–2016, and ongoing). In addition, continuous follow‐up for disease outcomes were available until March 31, 2019, using linkage to electronic health records of the UK National Health Service (NHS). Research ethics approvals were renewed at each contact; the most recent approval was from the University College London Hospital Committee on the Ethics of Human Research (reference number 85/0938). All participants gave written informed consent at each contact.

### 2.2 CVD

CVD included coronary heart disease, stroke, atrial fibrillation, or heart failure. We used three methods to ascertain cases of CVD, as done previously.^19^, ^20^ First, using linkage to the Hospital Episodes Statistics (HES) database, a national database that contains information on inpatient and outpatient care, using ICD (International Classification of Diseases) codes for coronary heart disease (ICD‐9 codes 410‐414 and ICD‐10 codes I20‐I25), stroke (ICD‐9 codes 430, 431, 434, 436 and ICD‐10 codes I60‐I64), atrial fibrillation (ICD‐9 code 427.3 and ICD‐10 code I48), and heart failure (ICD‐9 code 428 and ICD‐10 code I50). Second, coronary heart disease and atrial fibrillation were ascertained by 12‐lead resting electrocardiography using the Minnesota classification system. Third, stroke was ascertained by the World Health Organization Multinational Monitoring of Trends and Determinants in CVD (MONICA‐)‐Augsburg stroke questionnaires.

### 2.3 Incident dementia

We used three national registers (HES database, the Mental Health Services Data Set, and the Mortality Register) to ascertain dementia cases using ICD‐10 codes F00‐F03, F05.1, G30, and G31. The sensitivity of dementia using the HES database is 78% and the specificity is 92%.^21^ The Mental Health Services Data Set is a national database containing information on dementia for people in contact with mental health services in hospitals, outpatient clinics, and the community. Cause‐specific mortality data were drawn from the NHS national mortality register. Date of dementia was set at the first record of dementia diagnosis using all three databases.

### 2.4 Lifestyle factors

Four lifestyle factors were selected based on recommendations in current clinical guidelines from the European Society of Cardiology and the American Heart Association^12^, ^13^, ^14^ and were defined as being at recommended levels using body mass index (cutoff value, <30 kg/m^2^), smoking (not‐smoker), physical activity (cutoff value, ≥150 min/week of moderate‐to‐vigorous physical activity or ≥150 min/week of moderate physical activity or ≥75 min/week of vigorous physical activity), and diet (fruit or vegetables consumption ≥twice/day and high‐fiber bread consumption). Measurement of the lifestyle factors is described in Supplementary Methods 1.

### 2.5 Covariates

Covariates included sociodemographic measures, hypertension, and diabetes. Sociodemographic variables were assessed by self‐administered questionnaire and included age, sex, race/ethnicity (White, non‐White), marital status (married/cohabiting, other), and socioeconomic status using education (≤partially secondary school (age <16), higher secondary school (age <18), and ≥university). Hypertension was defined as a blood pressure of ≥140/90 mmHg or use of an antihypertensive medication. Type 2 diabetes was defined as a fasting glucose of ≥7 mmol/L at the clinical examination (available from 1997 onwards), use of diabetes medication, self‐reported diabetes, or record of diabetes in the HES database (ICD‐10 code E11).

### 2.6 Statistical analysis

The analyses were conducted in two steps.

#### 2.6.1 Association of CVD with incident dementia

We examined the association between CVD and incident dementia using Cox proportional hazards regression. Participants were censored at the date of record of dementia, death, or March 31, 2019, whichever came first. Participants who died over the follow‐up were censored at date of death to account for competing risk of death using cause‐specific hazard models.^22^ The proportional hazards assumption was examined using Schoenfeld's test. All analyses were adjusted for age (timescale), sex, race/ethnicity, education, marital status, and birth cohort in 5‐year age groups.

The analyses described above were undertaken using CVD status (yes/no) at 55, 60, 65, and 70 years, extracted using the total span of the study. For these analyses, start of follow‐up was the age at CVD status (55, 60, 65, and 70 years, respectively) and the hazard ratio (HR) and 95% confidence interval (CI) of risk of dementia were calculated by comparing those with and without CVD at age 55, 60, 65, and 70 years, respectively, in four sets of analyses. Subsequent analyses at age 60, 65, and 70 years involved use of 5‐year age bands for the onset of CVD. For example, at age 70 years, the group defined as having no CVD at age 70 was compared to a group diagnosed with CVD between 66 and 70 years, 61 and 65 years, and ≤60 years. We used a test for trend with age at onset categories as a linear variable to examine whether younger age at CVD onset was associated with an increased risk of dementia over the follow‐up. In additional analysis, we tested whether associations differed by sex and race/ethnicity but found no differences (all *P‐*values for interaction >0.05). Therefore, results are reported without stratification for these factors.

#### 2.6.2 Role of lifestyle factors for subsequent risk of dementia in participants with CVD

Among participants who developed CVD over the span of the study, we examined the association between the number of lifestyle factors at recommended levels (i.e., not‐smoking, guideline‐recommended levels of body mass index, physical activity, and diet) and incident dementia using Cox regression with age as the timescale. Data on lifestyle factors were extracted from the wave following CVD diagnosis for each participant with a CVD event, using all available data. For example, for a participant with incident CVD between the 1991–1993 and 1997–1999 waves of data collection, their data on lifestyle factors at the 1997–1999 wave (post‐CVD) were used in these analyses. In these analyses, participants were followed from the measurement of lifestyle factors until the date of record of dementia, death, or March 31, 2019, whichever came first. We examined the shape of the association between the number of lifestyle factors at recommended levels on a continuous scale (score 0–4) with risk of dementia using restricted cubic spline models with 4 Harrel knots,^23^ with score 3 (median number of lifestyle factors at recommended levels) as the reference. We then examined the association of the number of lifestyle factors at recommended levels, modeled as a linear variable, to examine the risk reduction per additional lifestyle factor at recommended levels. Finally, we examined the association of each of the four lifestyle factors at recommended levels with incidence of dementia in separate models. These analyses were adjusted for age (timescale), sex, race/ethnicity, education, marital status, and birth cohort in 5‐year groups (model 1); and additionally, for hypertension and diabetes (model 2). Analyses with the individual lifestyle factors as the determinant were additionally mutually adjusted (model 3).

We undertook further analyses to test the robustness of the main results. (1) Analysis was repeated after the consecutive exclusion of one lifestyle factor from the lifestyle factor score. (2) To minimize potential confounding effects by diabetes, we repeated the analysis excluding participants with prevalent type 2 diabetes (i.e., type 2 diabetes prior to or at CVD diagnosis). (3) Given that lifestyle factors levels might be affected by time between CVD diagnosis and the measure of lifestyle factors, analysis was repeated excluding participants where this period was >6 years. (4) We tested for differences in association of lifestyle factors with incident dementia as a function of sex and race/ethnicity. (5) We used inverse probability weighting to account for missing data on lifestyle factors.^24^ This involved calculation of the probability of being included in the analytical sample by using data on the four lifestyle factors at recommended levels at recruitment, sociodemographic factors, morbidities (coronary heart disease, stroke, atrial fibrillation, heart failure, hypertension, and diabetes) and dementia and mortality over the follow‐up, and stepwise‐selected interactions between covariates. The inverse of these probabilities was used as weights in Cox regression.

Statistical analyses were performed with Stata 15.1 (Statacorp) software. A two‐sided *P‐*value of <.05 was considered statistically significant.

## 3 RESULTS

The study population consisted of 10,216 participants who had complete data on covariates and were free of dementia at baseline in 1985–1988 (Figure S1). Mean age at the end of follow‐up was 74.7 years (SD 7.2) and 32.9% of participants were women (Table 1). Figure S1 also shows the derivation of the study populations at age 55, 60, 65, and 70 years. After a median follow‐up of 31.6 years (25th, 75th percentile: 31.1, 32.6), 650 participants (6.4%) had incident dementia; these participants were more often female and were more likely to be less educated and to have cardiometabolic co‐morbidities (Table 1). In total, 3275 (32.1%) participants developed CVD, of whom 258 (8.5%) were subsequently diagnosed with dementia. Mean age at CVD diagnosis was 65.4 (SD: 10.9) years, and 76.7 (SD: 6.0) at dementia diagnosis.

**TABLE 1:** Characteristics of the total study population, and according to dementia status.

		Dementia status at the end of follow‐up^a^, March 2019	
	Total study population (*n* = 10,216)	No dementia (*n* = 9566, 93.6%)	Dementia (*n* = 650, 6.4%)
**Characteristics at the end of follow‐up**			
Age at the end of follow‐up, years	74.7 (7.2)	74.6 (7.2)	76.7 (6.0)
Female sex, n (%)	3361 (32.9)	3089 (32.3)	272 (41.9)
Married/cohabiting, n (%)	7568 (74.1)	7108 (74.3)	190 (29.2)
Education			
≤Partial secondary school, n (%)	4848 (47.5)	4462 (46.6)	386 (59.4)
High school diploma,n (%)	2714 (26.6)	2578 (27.0)	136 (20.9)
≥University degree,n (%)	2654 (26.0)	2526 (26.4)	128 (19.7)
Race/ethnicity			
White, n (%)	9181 (89.9)	8626 (90.2)	555 (85.4)
Non‐White, n (%)	1035 (10.1)	940 (9.8)	95 (14.6)
Cardiovascular disease,n (%)	3275 (32.1)	2917 (30.5)	358 (55.1)
Coronary heart disease, n (%)	2290 (22.4)	2075 (21.7)	215 (33.1)
Stroke, n (%)	511 (5.0)	403 (4.2)	108 (16.6)
Atrial fibrillation, n (%)	1343 (13.2)	1.149 (12.0)	194 (29.9)
Heart failure, n (%)	635 (6.2)	531 (5.6)	104 (16.0)
Type 2 diabetes at the end of follow‐up, n (%)	1663 (16.3)	1497 (15.7)	166 (25.6)
Hypertension at the end of follow‐up, n (%)	6730 (65.9)	6201 (64.8)	529 (81.4)
**Lifestyle factors at age 60** ^b^	*n* = 7940	*n* = 7444	*n* = 496
Body mass index, kg/m^2^	26.3 (4.2)	26.3 (4.3)	26.3 (4.3)
Body mass index, <30 kg/m^2^, n (%)	6647 (83.7)	6235 (83.8)	412 (83.1)
Not‐smokers, n (%)	6974 (87.8)	6551 (88.0)	423 (85.3)
Moderate‐to‐vigorous physical activity, min/week	165 (56, 315)	167 (56, 315)	122 (36, 300)
Moderate‐to‐vigorous physical activity, ≥150 min/week, n (%)	4223 (53.2)	3978 (53.4)	245 (49.4)
Dietary habits at optimal level^c^, n (%)	1977 (24.9)	1.892 (25.4)	85 (17.1)
Number of behavioral risk factors at recommended level	3 (2, 3)	3 (2, 3)	3 (2, 3)

*Note*: Data are mean (SD) or median (25th, 75th percentile).

^a^ Median (25th percentile, 75th percentile) overall follow‐up was 31.6 (31.1, 32.6) years; for participants without dementia, it was 31.8 (31.1, 32.7) years and for participants with dementia it was 27.5 (24.6, 30.2) years at end of follow‐up.

^b^ Data on lifestyle factors at age 60 not shown for 2276 participants, 6 were excluded due to dementia diagnosis before age 60, 341 died before age 60, and 1929 had missing data on lifestyle factors.

^c^ Dietary habits at recommended level was defined as consumption of fruit and vegetable ≥twice/day and high‐fiber bread consumption.

### 3.1 Association of CVD with incident dementia

The association of prevalent CVD at age 55, 60, 65, and 70 years with dementia risk is shown in Table 2. Results show that early (i.e., ages 55, 60, and 65) onset of CVD was associated with a higher risk of dementia. At age 55, participants with CVD had a higher HR of incident dementia compared to participants without CVD (HR 1.52, 95% CI 1.09–2.13). This was also the case for prevalent CVD at age 60 (HR 1.59, 95% CI 1.23–2.05) and 65 (HR 1.47, 95% CI 1.18–1.82). Late‐onset CVD, reflected here as prevalent CVD at age 70, was not associated with incident dementia (HR 1.19, 95% CI 0.97–1.47).

**TABLE 2:** Association between CVD and incidence of dementia according to age at CVD^a^ onset.

	Total/dementia cases	Rate/1000 person‐years	Hazard ratio (95% CI)
**At age 55 years (median (25th, 75th percentile) follow‐up 19.6 (15.6, 25.1) years)**			
No history of cardiovascular disease	9532/613	3.18	Reference
History of cardiovascular disease	518/36	3.82	1.52 (1.09, 2.13)
**At age 60 years (median (25th, 75th percentile) follow‐up 14.8 (10.8, 20.2) years)**			
No history of cardiovascular disease	9026/577	4.10	Reference
Diagnosis 56–60 years	347/32	6.20	1.67 (1.17, 2.39)
Diagnosis ≤55 years	496/35	5.09	1.53 (1.08, 2.15)
*P* for linear trend			0.001
History of cardiovascular disease at age 60	843/67	5.56	1.59 (1.23, 2.05)
**At age 65 years (median (25th, 75th percentile) follow‐up 10.1 (6.1, 15.4) years)**			
No history of cardiovascular disease	8349/527	5.78	Reference
Diagnosis 61–65 years	434/33	7.07	1.23 (0.87, 1.75)
Diagnosis 56–60 years	335/32	9.28	1.75 (1.22, 2.50)
Diagnosis ≤55 years	465/33	7.37	1.52 (1.07, 2.17)
*P* for linear trend			<0.001
History of cardiovascular disease at age 65	1234/98	7.78	1.47 (1.18, 1.82)
**At age 70 years (median (25th, 75th percentile) follow‐up 6.7 (3.0, 11.4) years)**			
No history of cardiovascular disease	6375/438	9.13	Reference
Diagnosis 66–70 years	504/36	9.44	1.02 (0.72, 1.43)
Diagnosis 61–65 years	346/22	8.26	0.90 (0.59, 1.39)
Diagnosis ≤60 years	641/55	13.08	1.56 (1.18, 2.08)
*P* for linear trend			0.015
History of cardiovascular disease at age 70	1491/113	10.58	1.19 (0.97, 1.47)

*Note*: All analyses are adjusted for age (as the timescale), sex, race/ethnicity, education, marital status, and birth cohort (5‐year age groups).

Abbreviations: CI, confidence interval; CVD, cardiovascular disease.

^a^ CVD was defined as coronary heart disease, stroke, atrial fibrillation, or heart failure.

Analyses to examine the role of age at CVD onset in the risk of dementia using a test for linear trend, also shown in Table 2, suggest that much of the excess risk of dementia is due to CVD events before age 60. For example, in the analysis of CVD status at age 70 using participants without CVD at age 70 as the reference, participants with incident CVD between 66 and 70 years (HR 1.02, 95% CI 0.72–1.43) or 61 and 65 years (HR 0.90, 95% CI 0.59–1.39) did not have higher risk of dementia but those with CVD onset before 60 years had a higher risk of subsequent dementia (HR 1.56, 95% CI 1.18–2.08).

### 3.2 Role of lifestyle factors for subsequent risk of dementia in participants with CVD

**FIGURE 1:**
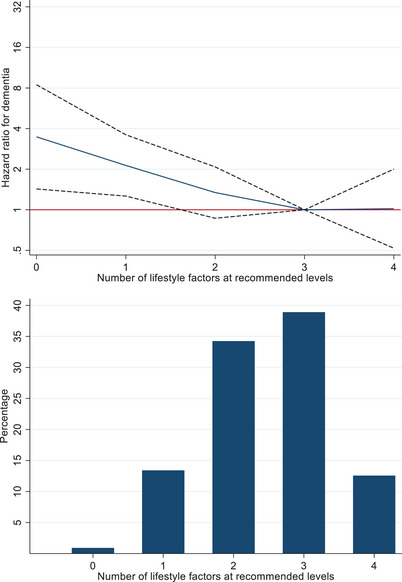
Lifestyle factors at recommended levels and incidence of dementia in participants with cardiovascular disease (CVD). NOTE. Hazard ratios (HRs) (95% confidence intervals [Cis]) are adjusted for age (timescale), sex, race/ethnicity, education, marital status, birth cohort (5‐year groups), and time between CVD diagnosis and measure of lifestyle factors; a score of 3 (median) was the reference (HR = 1).

Of the 3275 participants who developed CVD over the 31.6‐year span of the study, we had data on the four lifestyle factors after CVD diagnosis on 1536 participants (Figure S1). After a median follow‐up of 10.9 years (25th, 75th percentile: 6.4, 17.7), 115 (7.5%) of them had incident dementia. Mean age at CVD diagnosis was 61.2 years (SD 10.5) and 22.5% were female (Table S1). The 391 participants with CVD excluded from these analyses due to missing data on lifestyle factors post‐CVD were less educated, more often had type 2 diabetes and hypertension prior to or at CVD onset, and had poorer lifestyle factors compared to those included in the analyses (Table S2). As shown in Figure 1 there was a linear association between the number of lifestyle factors at recommended levels (continuous scale) and the risk of dementia (*p* for non‐linearity 0.49). In this group of persons with CVD, a greater number of lifestyle factors at recommended levels was associated with a lower risk of dementia (HR per additional lifestyle factor at recommended levels 0.73; 95% CI 0.59–0.92, Table 3). Of the individual lifestyle factors, being a not‐smoker and being physically active were associated with a lower HR of dementia, whereas guideline‐recommended levels of body mass index and dietary habits did not have an independent association with dementia.

**TABLE 3 alz13562-tbl-0003:** Association between lifestyle factors^a^ and incidence of dementia in participants with CVD.

Lifestyle factor factor^a^	Total/dementia cases	Hazard ratio (95% CI) Model 1^b^	Hazard ratio (95% CI) Model 2^c^	Hazard ratio (95% CI) Model 3^d^
**Lifestyle factor score (0 to 4)** Risk for one additional lifestyle factor at recommended levels	1536/115	0.74 (0.59, 0.92)	0.73 (0.59, 0.92)	N/A
**Individual lifestyle factors**				
**Body mass index**				
Not at recommended level	342/28	Reference	Reference	Reference
At recommended level	1194/87	0.71 (0.46, 1.11)	0.71 (0.46, 1.10)	0.72 (0.47, 1.13)
**Smoking**				
Not at recommended level	115/11	Reference	Reference	Reference
At recommended level	1421/104	0.51 (0.27, 0.98)	0.51 (0.27, 0.98)	0.53 (0.28, 1.02)
**Physical activity**				
Not at recommended level	740/71	Reference	Reference	Reference
At recommended level	796/44	0.60 (0.40, 0.89)	0.59 (0.40, 0.88)	0.61 (0.41, 0.91)
**Diet**				
Not at recommended level	1126/87	Reference	Reference	Reference
At recommended level	410/28	1.03 (0.67, 1.59)	1.03 (0.66, 1.59)	1.11 (0.71, 1.72)

Abbreviations: CI, confidence interval; CVD, cardiovascular disease; MPA, moderate physical activity; MVPA, moderate and vigorous physical activity; N/A, not applicable; VPA, vigorous physical activity.

^a^ Lifestyle factors measured after CVD diagnosis; body mass index at recommended level if <30 kg/m^2^; smoking at recommended level if not‐smoker; physical activity at recommended level if ≥150 min/week of MPA OR ≥75 min/week of VPA OR ≥150 min/week of MVPA; diet at recommended level if fruit and vegetable consumption ≥twice/day AND high‐fiber bread consumption.

^b^ Model 1 includes age (timescale), sex, race/ethnicity, education, marital status, birth cohort (5‐year groups), time between CVD diagnosis, and measure of lifestyle factors.

^c^ Model 2: model 1 + hypertension, and diabetes.

^d^ Model 3: model 2 + mutual adjustment for lifestyle factors.

### 3.3 Additional analyses

The results remained broadly similar when we removed body mass index, smoking, or diet from the continuous lifestyle score. After removing physical activity, the association between number of lifestyle factors and incident dementia was no longer statistically significant (HR 0.80, 95% CI 0.60–1.06), Table S3). Excluding participants with prevalent diabetes (*n* = 133, Table S4) or participants whose lifestyle factors were assessed >6 years after CVD diagnosis (*n* = 114, Table S5) yielded results consistent with those obtained in the main analysis. There was no evidence of sex differences in these analyses (all *P‐*values for interaction >.10; Table S6), although there was some evidence of race/ethnicity differences in the number of lifestyle factors and body mass index at recommended levels (*P*‐values for interaction .028 and .033, respectively; Table S6). Among participants of White ethnicity, the HR for dementia was 0.65 (95% CI 0.51–0.83) per additional lifestyle factor at recommended levels (Table S7). In addition, all individual lifestyle factors, except guideline‐recommended levels of dietary habits, were associated with incident dementia among participants of White ethnicity (Table S7). Analysis using inverse‐probability weighting to account for missing data on the lifestyle factors led to results that were consistent with those in the main analysis (Table S8).

## 4 DISCUSSION

The main finding of this study on 10,216 participants of the Whitehall II study using data spanning three decades is that onset of CVD in midlife but not in late life is associated with higher risk of dementia. There was no evidence of a dose–response relationship between age at CVD onset and the risk of dementia; the association between CVD and incident dementia appears to be driven by individuals with CVD before age 60. We also found that in individuals with CVD the risk of dementia is lower if a greater number of lifestyle factors, measured after the CVD event, are at recommended levels. To address the burden of old‐age dementia due to increases in life expectancy, our findings highlight the importance of prevention of early‐onset CVD, and lifestyle modification as a secondary prevention mechanism in those with CVD.

### 4.1 Comparison with previous studies

To date, few studies have examined the association of age at CVD onset (or time lived with CVD) with incident dementia. Most of the evidence comes from studies on atrial fibrillation. In four previous prospective population‐based studies^20^, ^25^, ^26^, ^27^ the risk of dementia was stronger in those with younger age at onset of atrial fibrillation (age ≤70 vs >70 in the Whitehall II study^20^ and the Intermountain Heart Collaborative Study,^26^ <67 vs ≥67 in the Rotterdam Study,^25^ and ≤57 vs >57 in the Atherosclerosis Risk in Communities (ARIC) Study^27^). Previous research on the association of coronary heart disease and stroke with cognitive outcomes have focused on cognitive decline rather than dementia. For example, a previous report of the Whitehall II study^28^ found a trend suggesting that longer duration of coronary heart disease was associated with faster cognitive decline, whereas in the Honolulu Heart program, longer duration of stroke or coronary heart disease was not associated with cognitive decline.^29^ We extend the results of these studies with a careful analysis of age at CVD onset at 55, 60, 65, and 70 years to show a graded association between age at CVD onset and risk of dementia.

We are not aware of studies that examined whether the risk of CVD‐associated dementia can be mitigated by modification or control of lifestyle factors. The exception being one previous study using data from the Korean National health Insurance database of 199,952 participants reporting that in patients with atrial fibrillation the risk of dementia over a median follow‐up of 3.4 years was lower if a greater number of lifestyle factors (i.e., smoking, alcohol consumption, and physical activity) were within the recommended levels.^17^ Furthermore, randomized trials on the effects of lifestyle factor modification in individuals with CVD with dementia or cognitive decline are scarce and limited by small numbers. Two relatively small trials from Norway (*n* = 195, 12‐month intervention)^30^ and Austria (*n* = 101, 24‐month intervention)^31^ evaluated the effect of multidomain lifestyle interventions among participants with a history of stroke and did not find beneficial effects on cognitive function. When these two studies were combined in a pooled data analysis, there was no beneficial effect on executive function or memory but there were indications for a beneficial effect on processing speed.^32^ These results need to be interpreted with caution, as the effect on processing speed was found only after imputation of drop‐outs, and not after full imputation or in the complete case analysis.

Subgroup analyses in trials on persons with CVD also showed no beneficial effects on cognitive function or dementia risk. For example, in the subgroup of participants with CVD (*n* = 1193, 35.0% of the total study sample) in The Prevention of Dementia by Intensive Vascular care (preDIVA) trial, a multidomain intervention based on smoking habits, diet, exercise, weight loss, and vascular risk management compared with the control intervention (regular cardiovascular risk management), found no beneficial effect on dementia risk after 6 years of follow‐up.^33^ In addition, in 244 participants with CVD (20.7% of the total study sample) in the 2‐year Finnish Geriatric Intervention Study to Prevent Cognitive Impairment and Disability (FINGER) there was no statistically significant difference in cognitive decline between those who underwent a multidomain lifestyle intervention (diet, exercise, cognition, and vascular risk management) compared to the control group (regular health advice).^34^

The present study found that in individuals with CVD the risk of dementia is lower if a greater number of lifestyle factors are at recommended levels. Apart from dietary habits, all individual lifestyle factors at recommended levels were associated with a lower incidence of dementia, with the associations being statistically significant in analyses of participants of White ethnicity. The findings on diet may have several explanations. First, there may be no causal relationship between diet quality and risk of dementia and the Lancet Commission on Dementia did not include diet in the list of modifiable risk factors associated with dementia.^35^ Second, dietary behavior is multidimensional and consists of intake of various food groups, beverages, and nutrients. In this study, guideline‐recommended levels of dietary habits were based on consumption of fruit or vegetables and bread, which may not reflect all aspects of a healthy diet. Further research using a more comprehensive measure of dietary habits in participants with CVD is needed to better understand the effect of diet on dementia in this group.

### 4.2 Pathophysiological mechanisms

The pathophysiological mechanisms underlying the association of vascular risk factors and disease with dementia unfold over a long preclinical period and are likely to involve multiple aetiologies. Dementia in CVD may be a consequence of structural cerebrovascular damage due to stroke^36^ or cerebral (micro)emboli associated with atrial fibrillation^10^ or heart failure,^8^ and chronic cerebral hypoperfusion due to reduced cardiac output associated with coronary heart disease,^8^ atrial fibrillation,^10^ or heart failure.^8^ In addition, atherosclerotic processes in CVD are associated with cerebral microvascular dysfunction and damage, which in turn may lead to dementia.^36^ Other biological mechanisms underlying CVD‐related dementia may include low‐grade inflammation and neurodegeneration.^36^ The lifestyle factors examined in our study have each been associated with one or more of these etiologies.^36^, [Table alz13562-tbl-0003]

### 4.3 Clinical implications

In addition to previous evidence suggesting that cardiometabolic risk factors and disease^5^, ^7^ and lifestyle factors^15^ might need to be targeted as early as in midlife to prevent dementia at older ages, the current study suggests that this might also be the case for people with CVD. In addition, the present study suggests that after onset of CVD, multifactorial lifestyle modification might potentially reduce the risk of dementia, as previously also shown in studies based on the general population.^15^, ^16^ Taken together, these findings suggest that in addition to lowering the risk of recurrent CVD events and mortality, reducing risk of dementia may be an additional motivation for CVD patients to adopt a healthy lifestyle. Of the selected lifestyle factors, physical activity appears to be a particularly important target to prevent dementia in individuals with CVD. In the general population, however, there is inconsistent evidence on the role of physical activity in dementia risk in previous observational^37^ and intervention studies.^38^ It is possible that these differences are due to CVD‐related dementia being primarily vascular dementia while much of the dementia in the general population is due to Alzheimer's disease.^8^ Lack of data on dementia sub‐types did not allow further investigation of this issue in the present study. It is worth noting that trials that solely target physical activity in participants with CVD with dementia or cognitive performance as the outcome are scarce and that the existing evidence does not show beneficial effects.^39^ The limited follow‐up in such studies is a concern, particularly for dementia. It is also possible that interventions in those with CVD need to be multi‐domain, and include a wider range of lifestyle factors such as sleep,^40^, ^41^ social isolation,^42^ depressive symptoms,^43^ and air population.^44^

### 4.4 Strengths and limitations

The key strengths of this study include the large sample size and a long follow‐up for CVD and dementia, which allowed examination of the association of CVD in both midlife and late life with incident dementia. Furthermore, the study design consisted of repeated clinical examinations to study lifestyle factors measured after CVD diagnosis. The findings need to be considered in light of study limitations. First, the observational design precludes causal conclusions, and the results are not comparable to interventions, as lifestyle factors at recommended levels in our study could be naturally at recommended levels or following behavioral modification. Second, we did not have the statistical power to examine whether the associations with dementia differed according to the types of CVD. Third, data on lifestyle factors and medication were self‐reported. Fourth, use of linkage to electronic health records for ascertainment of CVD and dementia cases may lead to some bias as individuals with milder forms of disease may not seek medical care.^21^, ^45^ It is also possible that CVD patients may be more likely to be diagnosed with dementia due to more frequent contact with medical professionals for the management of CVD. However, electronic health records used in this study for ascertainment of dementia cases contain information on both health and social care and have national coverage to improve the sensitivity of this method of ascertainment. In addition, electronic health records have been used in the UK to develop risk‐prediction models for CVD that have been externally validated and are currently used in clinical practice.^46^, ^47^ Fifth, we were unable to examine whether the role of lifestyle factors in individuals with CVD differed as a function of age at assessment of lifestyle factors due to a small number of participants in these analyses. Sixth, participants excluded from the analysis on lifestyle factors post‐CVD and incident dementia due to missing data on lifestyle factors had an adverse profile, suggesting that selection bias may have reduced our ability to detect associations. Seventh, participants of the Whitehall II study were all employed at study recruitment (1985) and are likely to be healthier than the general population. However, this does not necessarily affect risk factor–disease associations.^48^ A further limitation is that participants were mostly of White ethnicity and the extent to which the results can be generalized to other groups remains unclear. There was some evidence that our findings might not apply to other race/ethnicity groups, but we could not examine associations in participants of non‐White ethnicity due to small numbers.

### 4.5 Conclusions

The current study with a median follow‐up of 31.6 years shows that CVD is associated with a higher risk of dementia when onset of CVD is in midlife (before age 60) but not when onset is in late life. These findings highlight the need for public health policies for prevention and management of CVD in midlife or earlier, to mitigate the risk of dementia at older ages. In addition, this study shows that the risk of dementia in participants with CVD is lower when a greater number of lifestyle factors are at recommended levels. These findings provide important evidence to encourage adoption of a healthy lifestyle in persons living with CVD.

## CONFLICT OF INTEREST STATEMENT

The authors declare no conflicts of interest. Author disclosures are available in the Supporting Information.

## Supporting information

Supporting Information

Supporting Information
