# Supplementary material for: Age at cardiovascular disease onset, dementia risk, and the role of lifestyle factors
Source: Alzheimers Dement. 2023 Dec 12;20(3):1693–702. doi: 10.1002/alz.13562 (PMC10947967; doi:10.1002/alz.13562)
Supplement: Supplementary file 1 — Supporting Information [file ALZ-20-1693-s002.pdf]

Age at cardiovascular disease onset, dementia risk, and the role of lifestyle factors

April C.E. van Gennip, Thomas T. van Sloten, Aurore Fayosse, Séverine Sabia, Archana Singh-Manoux

Supplementary material

Supplementary Methods

Supplementary Methods 1. Measurement of lifestyle factors

Supplementary Tables

Supplementary Table 1. Characteristics of participants with CVD, overall and according to dementia status

Supplementary Table 2. Characteristics of included participants with CVD, and those excluded due to missing data on lifestyle factors

Supplementary Table 3. Association between the lifestyle factor* score and incidence of dementia† in participants with CVD – consecutive exclusion of lifestyle factors

Supplementary Table 4. Association between lifestyle factors* and incidence of dementia† in participants with CVD – excluding participants with prevalent diabetes‡ (n=133)

Supplementary Table 5. Association between lifestyle factors* and incidence of dementia† in participants with CVD – excluding participants with lifestyle factors assessed more than 6 years after CVD (n=114)

Supplementary Table 6. Interaction with sex and race/ethnicity for the association of lifestyle factors* with incident dementia† in participants with CVD

Supplementary Table 7. Association between lifestyle factors* and incidence of dementia† in participants with CVD – restricting analysis to participants of white ethnicity (n=1,354)

Supplementary Table 8. Association of lifestyle factoral lifestyle factors* and incidence of dementia in participants with CVD – using inverse-probability weighting to account for missing data

Supplementary Figures

Supplementary Figure 1. Flowchart derivation of the study population

Supplementary Methods 1. Measurement of lifestyle factors

We used data collected at each of the clinical examinations (1985-1988, 1991-1993, 1997-1999, 2002-2004, 2007-2009, 2012-2013, and 2015-2016) to extract information on four lifestyle factors on the clinical examination following diagnosis of cardiovascular disease (CVD) for every participant. Smoking status, physical activity (hours of moderate-to-vigorous physical activity per week), and diet (fruit and vegetable consumption, consumption of high fibre bread) were assessed using questionnaires. A trained nurse used standardized protocols to measure body mass index (weight/height^2 in kg/m^2). Weight was measured in underwear to the nearest 0.1 kg on Soehnle electronic scales with digital readout (Leifheit, Nassau, Germany), and height was measured in bare feet to the nearest 1 mm by using a stadiometer with the participant standing erect with the head in the Frankfurt plane.

Supplementary Table 1. Characteristics of participants with CVD, overall and according to dementia status

	**All individuals with cardiovascular disease*** (n=1,536)	**Dementia status†**	
		**No dementia** (n=1,421, 92.5%)	**Dementia** (n=115, 7.5%)
**Characteristics following onset of cardiovascular disease**			
Age at onset of cardiovascular disease, years	61.2 (10.5)	61.1 (10.4)	62.3 (10.8)
Female sex, n (%)	345 (22.5)	311 (21.9)	34 (29.6)
Married/cohabiting, n (%)	1,173 (76.4)	1,094 (77.0)	79 (68.7)
Education			
≤Partial secondary school, n (%)	770 (50.1)	706 (49.7)	64 (55.7)
High school diploma, n (%)	402 (26.2)	384 (27.0)	18 (15.7)
≥University degree, n (%)	364 (23.7)	331 (23.3)	33 (28.7)
Race/ethnicity			
White, n (%)	1,354 (88.2)	1,259 (88.6)	95 (82.6)
Non-white, n (%)	182 (11.9)	162 (11.4)	20 (17.4)
Coronary heart disease, n (%)	1,231 (80.1)	1,136 (79.9)	95 (82.6)
Stroke, n (%)	187 (12.2)	154 (10.8)	33 (28.7)
Atrial fibrillation, n (%)	634 (41.3)	567 (39.9)	67 (58.3)
Heart failure, n (%)	243 (15.8)	214 (15.1)	29 (25.2)
Type 2 diabetes prior to or at CVD diagnosis, n (%)	133 (8.7)	124 (8.7)	9 (7.8)
Hypertension prior to or at CVD diagnosis, n (%)	850 (55.4)	789 (55.5)	61 (53.0)
**Lifestyle factors following onset of cardiovascular disease‡**			
Body mass index, kg/m^2	27.3 (4.5)	27.3 (4.5)	27.2 (4.5)
Body mass index, <30 kg/m^2, n (%)	1,194 (77.7)	1,107 (77.9)	87 (75.7)
Nonsmokers, n (%)	1,421 (92.5)	1,317 (92.7)	104 (90.4)
Moderate-to-vigorous physical activity, min/week	158 (38, 300)	161 (45, 300)	75 (0, 236)
Moderate-to-vigorous physical activity, ≥150 min/week, n (%)	796 (51.8)	652 (52.9)	44 (38.3)
Dietary habits at recommended level§, n (%)	410 (27.7)	382 (26.9)	28 (24.4)
Number of lifestyle factors at recommended levels	3 (2, 3)	3 (2, 3)	2 (2, 3)

NOTE. Data are means (SD) or median (25th percentile, 75th percentile).

* CVD was defined as coronary heart disease, stroke, atrial fibrillation, or heart failure.

† Median (25th percentile, 75th percentile) overall follow-up was 10.9 (6.4, 17.7) years, for participants without dementia it was 10.9 (6.4, 17.1) years and for participants with incident dementia it was 11.0 (5.3, 19.7) years.

‡ Lifestyle factors measured after diagnosis of CVD (median delay between CVD and lifestyle factors measurement: 2.3 years (1.5; 3.7).

§ Dietary habits at recommended level was defined as consumption of fruit and vegetable twice a day or more and consumption of high fibre bread.

Abbreviations: CVD, cardiovascular disease.

Supplementary Table 2. Characteristics of included participants with CVD, and those excluded due to missing data on lifestyle factors.

	**All participants with CVD*** (n=1,536)	**Participants with CVD* excluded due to missing data on lifestyle factors** (n=391)
**Characteristics following onset of cardiovascular disease**		
Age at onset of cardiovascular disease, years	61.2 (10.5)	62.3 (10.1)
Female sex, n (%)	345 (22.5)	117 (29.9)
Married/cohabiting, n (%)	1,173 (76.4)	289 (73.9)
Education		
≤Partial secondary school, n (%)	770 (50.1)	213 (54.5)
High school diploma, n (%)	402 (26.2)	104 (26.6)
≥University degree, n (%)	364 (23.7)	74 (18.9)
Race/ethnicity		
White, n (%)	1,354 (88.2)	327 (83.6)
Non-white, n (%)	182 (11.9)	64 (16.4)
Coronary heart disease, n (%)	1,231 (80.1)	314 (80.3)
Stroke, n (%)	187 (12.2)	64 (16.4)
Atrial fibrillation, n (%)	634 (41.3)	153 (39.1)
Heart failure, n (%)	243 (15.8)	82 (21.0)
Type 2 diabetes prior to or at CVD onset, n (%)	133 (8.7)	37 (9.5)
Hypertension prior to or at CVD onset, n (%)	850 (55.4)	235 (60.1)
**Lifestyle factors following onset of cardiovascular disease‡**		
Body mass index, kg/m^2	27.3 (4.5)	27.3 (17.6)
Body mass index, <30 kg/m^2, n (%)	1,194 (77.7)	93 (73.8)
Nonsmokers, n (%)	1,421 (92.5)	225 (88.6)
Moderate-to-vigorous physical activity, min/week	158 (38, 300)	86 (15; 240)
Moderate-to-vigorous physical activity, ≥150 min/week, n (%)	796 (51.8)	105 (38.5)
Dietary habits at recommended level†, n (%)	410 (27.7)	77 (28.2)

NOTE. Data are means (SD) or median (25th percentile, 75th percentile).

* CVD was defined as coronary heart disease, stroke, atrial fibrillation, or heart failure.

† Dietary habits at recommended level was defined as consumption of fruit and vegetable twice a day or more and consumption of high fibre bread.

Abbreviations: CVD, cardiovascular disease.

Supplementary Table 3. Association between the lifestyle factor* score and incidence of dementia† in participants with CVD – consecutive exclusion of lifestyle factors

Lifestyle factor*	Total/dementia cases	Hazard ratio (95% CI) Model 1‡	Hazard ratio (95% CI) Model 2§
**Number of lifestyle factors**			
Risk for one additional lifestyle factor at recommended levels removing body mass index	1,536/115	0.73 (0.56, 0.95)	0.72 (0.55, 0.95)
Risk for one additional lifestyle factor at recommended levels removing smoking	1,536/115	0.73 (0.56, 0.95)	0.72 (0.55, 0.95)
Risk for one additional lifestyle factor at recommended levels removing physical activity	1,536/115	0.80 (0.60, 1.06)	0.80 (0.60, 1.06)
Risk for one additional lifestyle factor at recommended levels removing diet	1,536/115	0.65 (0.50, 0.84)	0.64 (0.49, 0.83)

* Lifestyle factors measured after diagnosis of CVD; body mass index at recommended level if <30 kg/m2; smoking at recommended level if not-smoker; physical activity at recommended level if ≥150 min/week of MPA OR ≥75 min/week of VPA OR ≥150 min/week of MVPA; diet at recommended level if fruit and vegetable consumption ≥twice/day AND high fibre bread consumption.

† Median (25th percentile, 75th percentile) overall follow-up was 10.9 (6.5, 16.4) years, for participants without dementia it was 10.9 (6.5, 16.4) years and for participants with incident dementia it was 10.5 (5.6, 18.7) years at end of follow-up.

‡ Model 1 includes age (as the time scale), sex, race/ethnicity, education, marital status, birth cohort (5-year groups), and time between diagnosis of CVD and measure of lifestyle factors.

§ Model 2 includes variables in model 1 + hypertension and diabetes.

Abbreviations: CVD, cardiovascular disease; CI, confidence interval; MPA, moderate physical activity; VPA, vigorous physical activity; MVPA, moderate and vigorous physical activity.

Supplementary Table 4. Association between lifestyle factor* and incidence of dementia† in participants with CVD – excluding participants with prevalent diabetes‡ (n=133)

Lifestyle factor*	Total/ dementia cases	Hazard ratio (95% CI) Model 1§	Hazard ratio (95% CI) Model 2¶	Hazard ratio (95% CI) Model 3#
**Number of lifestyle factors**				
Risk for one additional lifestyle factor at recommended levels	1,403/106	0.77 (0.61, 0.97)	0.76 (0.60, 0.96)	N/A
**Individual lifestyle factors**				
**Body mass index**				
Not at recommended level	298/23	Reference	Reference	Reference
At recommended level	1,105/83	0.81 (0.50, 1.30)	0.80 (0.50, 1.29)	0.82 (0.51, 1.32)
**Smoking**				
Not at recommended level	108/11	Reference	Reference	Reference
At recommended level	1,295/95	0.48 (0.25, 0.93)	0.48 (0.25, 0.93)	0.50 (0.26, 0.97)
**Physical activity**				
Not at recommended level	739/41	Reference	Reference	Reference
At recommended level	664/65	0.60 (0.40, 0.91)	0.59 (0.39, 0.89)	0.61 (0.40, 0.92)
**Diet**				
Not at recommended level	1,026/79	Reference	Reference	Reference
At recommended level	377/27	1.08 (0.69, 1.69)	1.07 (0.68, 1.67)	1.15 (0.73, 1.81)

* Lifestyle factors measured after diagnosis of CVD; body mass index at recommended level if <30 kg/m^2; smoking at recommended level if not-smoker; physical activity at recommended level if ≥150 min/week of MPA OR ≥75 min/week of VPA OR ≥150 week of MVPA; diet if consumption of fruit and vegetable twice a day or more AND consumption of high fibre bread.
† Median (25th percentile, 75th percentile) overall follow-up was 11.1 (6.6, 20.1) years, for participants without dementia it was 11.1 (6.6, 20.1) years and for participants with incident dementia it was 11.8 (5.6, 20.0) years at end of follow-up.
‡ Prevalent diabetes was defined as type 2 diabetes prior to or at CVD diagnosis.
§ Model 1 includes age (as the time scale), sex, race/ethnicity, education, marital status, birth cohort (5-year groups), and time between diagnosis of CVD and measure of lifestyle factors.
¶ Model 2 includes variables in model 1 + hypertension and diabetes.
# Model 3 includes variables in model 2 + mutual adjustment for lifestyle factors.
Abbreviations: CVD, cardiovascular disease; CI, confidence interval; MPA, moderate physical activity; VPA, vigorous physical activity; MVPA, moderate and vigorous physical activity; N/A, not applicable.

Supplementary Table 5. Association between lifestyle factors* and incidence of dementia† in participants with CVD – excluding participants with lifestyle factors assessed more than 6 years after CVD diagnosis (n=114)

Lifestyle factor*	Total/ dementia cases	Hazard ratio (95% CI) Model 1‡	Hazard ratio (95% CI) Model 2§	Hazard ratio (95% CI) Model 3¶
**Lifestyle factor score (0 to 4)**				
Risk for one additional lifestyle factor at recommended levels	1,422/106	0.70 (0.56, 0.88)	0.69 (0.55, 0.87)	N/A
**Individual lifestyle factors**				
**Body mass index**				
Not at recommended level	328/28	Reference	Reference	Reference
At recommended level	1,094/78	0.66 (0.42, 1.03)	0.65 (0.42, 1.02)	0.67 (0.43, 1.05)
**Smoking**				
Not at recommended level	95/10	Reference	Reference	Reference
At recommended level	1,327/96	0.46 (0.23, 0.90)	0.46 (0.23, 0.90)	0.48 (0.24, 0.95)
**Physical activity**				
Not at recommended level	739/39	Reference	Reference	Reference
At recommended level	683/67	0.53 (0.35, 0.81)	0.52 (0.34, 0.79)	0.54 (0.36, 0.83)
**Diet**				
Not at recommended level	1,024/79	Reference	Reference	Reference
At recommended level	398/27	1.04 (0.67, 1.63)	1.04 (0.67, 1.63)	1.15 (0.73, 1.82)

* Lifestyle factors measured after diagnosis of CVD; body mass index at recommended level if <30 kg/m^2; smoking at recommended level if not-smoker; physical activity at recommended level if ≥150 min/week of MPA OR ≥75 min/week of VPA OR ≥150 week of MVPA; diet if consumption of fruit and vegetable twice a day or more AND consumption of high fibre bread.

† Median (25th percentile, 75th percentile) overall follow-up was 10.7 (6.2, 16.1) years, for participants without dementia it was 10.7 (6.3, 16.0) years and for participants with incident dementia it was 10.3 (5.3, 18.0) years at end of follow-up.

‡ Model 1 includes age (as the time scale), sex, race/ethnicity, education, marital status, birth cohort (5-year groups), and time between diagnosis of CVD and measure of lifestyle factors.

§ Model 2 includes variables in model 1 + hypertension and diabetes.

¶ Model 3 includes variables in model 2 + mutual adjustment for lifestyle factors.

Abbreviations: CVD, cardiovascular disease; CI, confidence interval; MPA, moderate physical activity; VPA, vigorous physical activity; MVPA, moderate and vigorous physical activity; N/A, not applicable.

Supplementary Table 6. Interaction of sex and race/ethnicity for the association of lifestyle factors* with incident dementia† in participants with CVD

	Sex	Race/ethnicity
	P value for interaction	
Body Mass Index	0.614	0.033‡
Smoking	0.989	0.330
Physical activity	0.687	0.965
Diet	0.600	0.130
Number of lifestyle factors at recommended levels	*0.334*	*0.028‡*

NOTE. All analyses adjusted for age (as the time scale), sex, race/ethnicity, education, marital status, birth cohort (5-year groups), and time between diagnosis of CVD and measure of lifestyle factors.

* Lifestyle factors measured after diagnosis of CVD; body mass index at recommended level if <30 kg/m^2; smoking at recommended level if not-smoker; physical activity at recommended level if ≥150 min/week of MPA OR ≥75 min/week of VPA OR ≥150 week of MVPA; diet if consumption of fruit and vegetable twice a day or more AND consumption of high fibre bread.

† Median (25th percentile, 75th percentile) overall follow-up was 10.9 (6.5, 16.4) years, for participants without dementia it was 10.9 (6.5, 16.4) years and for participants with incident dementia it was 10.5 (5.6, 18.7) years at end of follow-up.

‡ The statistically significant interaction suggests that the protective association of these factors were stronger in participants of white ethnicity. Results of the analysis restricted to those of white ethnicity are shown in Supplementary Table 7. We could not perform analyses restricted to those of non-white ethnicity, because of the relatively small number of participants and dementia cases in this group (n=182, of whom n=20 had incident dementia).

Abbreviations: CVD, cardiovascular disease. MPA, moderate physical activity; VPA, vigorous physical activity; MVPA, moderate and vigorous physical activity;.

Supplementary Table 7. Association between lifestyle factors* and incidence of dementia† in participants with CVD – restricting analysis to participants of white ethnicity (n=1,354)

Lifestyle factor*	Total/ dementia cases	Hazard ratio (95% CI) Model 1‡	Hazard ratio (95% CI) Model 2§	Hazard ratio (95% CI) Model 3¶
**Lifestyle factor score (0 to 4)**				
Risk for one additional lifestyle factor at recommended levels	1,354/95	0.65 (0.52, 0.83)	0.65 (0.51, 0.83)	N/A
**Individual lifestyle factors**				
**Body mass index**				
Not at recommended level	304/27	Reference	Reference	Reference
At recommended level	1,050/68	0.54 (0.34, 0.86)	0.54 (0.34, 0.85)	0.55 (0.34, 0.87)
**Smoking**				
Not at recommended level	101/10	Reference	Reference	Reference
At recommended level	1,253/85	0.42 (0.21, 0.83)	0.42 (0.21, 0.84)	0.45 (0.22, 0.89)
**Physical activity**				
Not at recommended level	607/55	Reference	Reference	Reference
At recommended level	747/40	0.60 (0.39, 0.91)	0.59 (0.39, 0.91)	0.63 (0.41, 0.96)
**Diet**				
Not at recommended level	969/71	Reference	Reference	Reference
At recommended level	385/24	0.91 (0.57, 1.45)	0.91 (0.57, 1.45)	0.99 (0.62, 1.60)

* Lifestyle factors measured after diagnosis of CVD; body mass index at recommended level if <30 kg/m^2; smoking at recommended level if not-smoker; physical activity at recommended level if ≥150 min/week of MPA OR ≥75 min/week of VPA OR ≥150 week of MVPA; diet if consumption of fruit and vegetable twice a day or more AND consumption of high fibre bread.

† In whites median (25th, 75th percentile) overall follow-up was 10.8 (6.3, 16.5) years, for participants without dementia it was 10.8 (6.4, 16.4) years and for participants with incident dementia it was 10.5 (5.2, 19.7) years at end of follow-up.

‡ Model 1 includes age (as the time scale), sex, education, marital status, birth cohort (5-year groups), and time between diagnosis of CVD and measure of lifestyle factors.

§ Model 2 includes variables in model 1 + hypertension and diabetes.

¶ Model 3 includes variables in model 2 + mutual adjustment for lifestyle factors.

Abbreviations: CVD, cardiovascular disease; CI, confidence interval; MPA, moderate physical activity; VPA, vigorous physical activity; MVPA, moderate and vigorous physical activity; N/A, not applicable.

Supplementary Table 8. Association of lifestyle factors* and incidence of dementia in participants with CVD – using inverse-probability weighting to account for missing data

Lifestyle factor*	Total/ dementia cases	Hazard ratio (95% CI) Model 1†	Hazard ratio (95% CI) Model 2‡	Hazard ratio (95% CI) Model 3§
**Lifestyle factor score (0 to 4)**				
Risk for one additional lifestyle factor at recommended levels	1,536/115	0.74 (0.56, 0.97)	0.74 (0.56, 0.97)	N/A
**Individual lifestyle factors**				
**Body mass index**				
Not at recommended level	342/28	Reference	Reference	Reference
At recommended level	1,194/87	0.68 (0.43, 1.08)	0.68 (0.43, 1.08)	0.70 (0.44, 1.10)
**Smoking**				
Not at recommended level	115/11	Reference	Reference	Reference
At recommended level	1,421/104	0.56 (0.28, 1.11)	0.56 (0.28, 1.11)	0.58 (0.29, 1.17)
**Physical activity**				
Not at recommended level	740/71	Reference	Reference	Reference
At recommended level	796/44	0.60 (0.40, 0.91)	0.59 (0.39, 0.90)	0.62 (0.41, 0.93)
**Diet**				
Not at recommended level	1,126/87	Reference	Reference	Reference
At recommended level	410/28	1.02 (0.64, 1.62)	1.02 (0.65, 1.61)	1.11 (0.70, 1.76)

* Lifestyle factors measured after diagnosis of CVD; body mass index at recommended level if <30 kg/m^2; smoking at recommended level if not-smoker; physical activity at recommended level if ≥150 min/week of MPA OR ≥75 min/week of VPA OR ≥150 min/week of MVPA; diet at recommended level if fruit and vegetable consumption ≥twice/day AND high fibre bread consumption.
† Model 1 includes age (as the time scale), sex, education, marital status, birth cohort (5-year groups), and time between diagnosis of CVD and measure of lifestyle factors.
‡ Model 2 includes variables in model 1 + hypertension and diabetes.
§ Model 3 includes variables in model 2 + mutual adjustment for lifestyle factors.
Abbreviations: CVD, cardiovascular disease; CI, confidence interval; MPA, moderate physical activity; VPA, vigorous physical activity; MVPA, moderate and vigorous physical activity; N/A, not applicable.

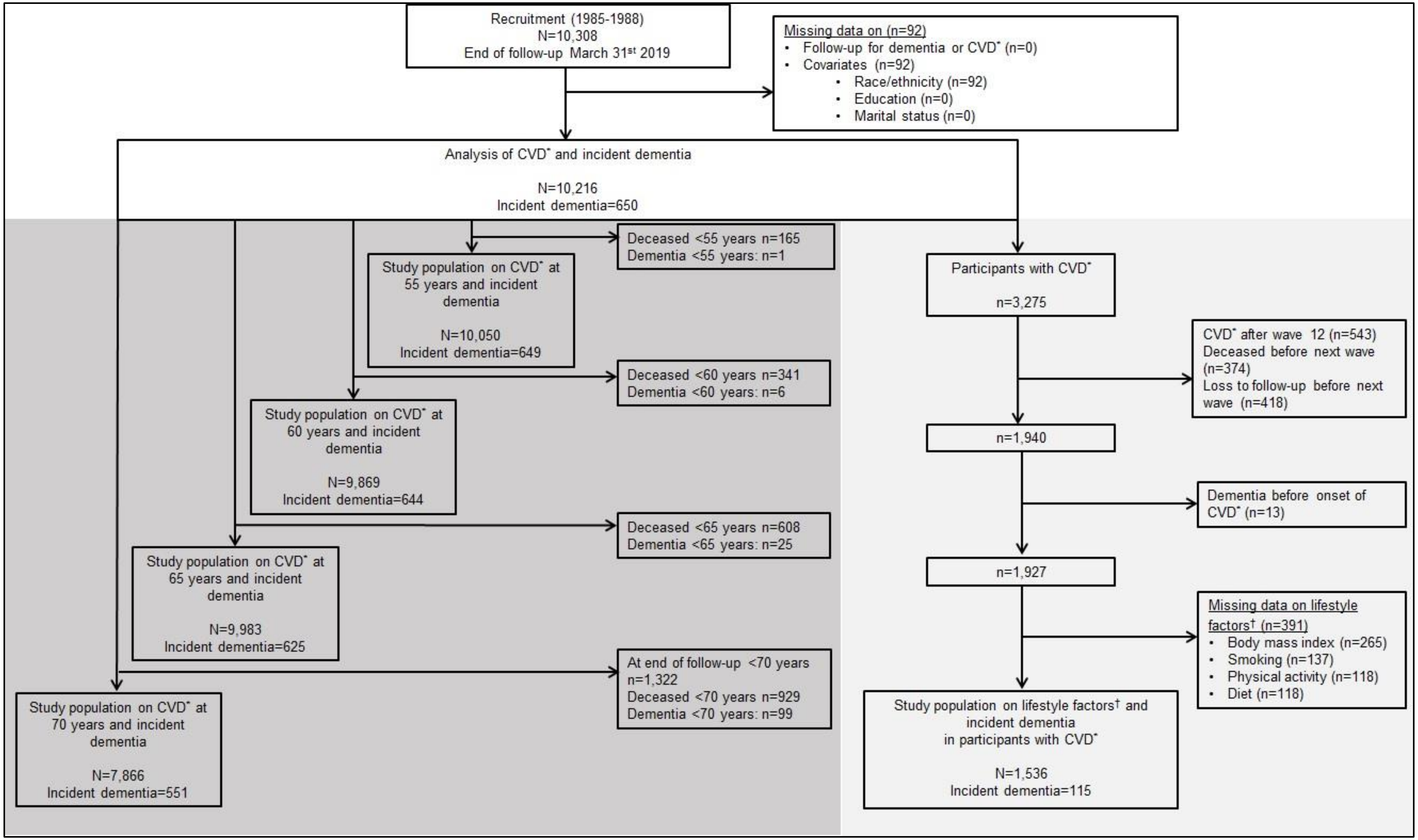

Supplementary Figure 1. Flowchart derivation of the study populations.

NOTE. Missing data not mutually exclusive.
* CVD was defined as coronary heart disease, stroke, atrial fibrillation, or heart failure. † Lifestyle factors measured after diagnosis of CVD.
Abbreviations: CVD, cardiovascular disease.
